# Tobacco smoking and health-related quality of life among university students: Mediating effect of depression

**DOI:** 10.1371/journal.pone.0227042

**Published:** 2020-01-08

**Authors:** Marija Milic, Tatjana Gazibara, Tatjana Pekmezovic, Darija Kisic Tepavcevic, Gorica Maric, Aleksandra Popovic, Jasmina Stevanovic, Karamchand Hukumchand Patil, Hagai Levine

**Affiliations:** 1 Braun School of Public Health and Community Medicine, Hebrew University-Hadassah, Jerusalem, Israel; 2 Department of Epidemiology, School of Medicine, University of Pristina temporarily seated in Kosovska Mitrovica, Kosovska Mitrovica, Serbia; 3 Institute of Epidemiology, School of Medicine, University of Belgrade, Belgrade, Serbia; 4 School for Sports and Physical Education, University of Belgrade, Belgrade, Serbia; University of Calfornia San Francisco, UNITED STATES

## Abstract

The aim of this study was to assess the association between cigarette smoking and health-related quality of life (HRQoL) among students in two different universities, and the potential mediating effect of depression. Participants were students who came for mandatory check-ups at Student Health Care Centers in two Universities in Serbia, differing by socio-politically and economically environments. Students completed socio-demographic questionnaire, Beck Depression Inventory (BDI) and the SF-36 questionnaire for assessment of HRQoL. In both populations, after adjustment for socio-demographic, behavioral and health factors, smoking was associated with poorer Mental Composite Score (MCS) and Physical Composite Score (PCS) (Belgrade 1,624 students: MCS β = 3.38, 95% confidence interval [CI] 1.31, 5.44, PCS β = 1.01, 95% CI -0.50, 2.52; Kosovska Mitrovica 514 students: MCS β = 5.06, 95% CI 1.74, 8.37, PCS β = 3.29, 95% CI 0.75, 5.83). After additional adjustment for BDI score, the observed associations were lost (Belgrade: MCS β = 1.12, 95% CI -0.57, 2.80, PCS β = -0.40, 95% CI -1.71, 0.92; Kosovska Mitrovica: MCS β = 0.77, 95% CI -2.06, 3.60, PCS β = 0.56, 95% CI -1.75, 2.87). Higher BDI score was associated with poorer PCS and MCS across all quintiles. The association of smoking with impairment of HRQoL among university students in two different settings was mediated by higher levels of depressive symptoms. These findings highlight the need for further research on the interaction between smoking, mental health and quality of life, with implications for prevention, diagnosis and treatment.

## Introduction

Smoking and exposure to tobacco smoke are a major risk factor of morbidity and mortality worldwide [1]. Evidence suggests that nearly 9 out of 10 current smokers initiated smoking before adulthood, and 98% of smokers first experience tobacco smoking until 26 years of age [2].

Some findings indicate that depression might be the strongest factor contributing to smoking initiation and substance use among university students [3]. At a global level, depression has been considered as a common mental health disorder among University students [4]. Conversely, in this particular population, smoking has been reported as a predisposing factor for the development of depressive symptoms [5, 6]. Depression is suggested to occur two times more frequently among current moderate smokers compared to non-smokers [7]. While reciprocal association between smoking and depression has previously been observed, the directionality of this association has not yet been addressed [8, 9]. However, a recent study found strong evidence to support a hypothesis that smoking could be a risk factor for development of depression for both lifetime smoking and smoking initiation [10].

Previous studies reported that, compared with current smokers, persons who had never smoked had significantly better health-related quality of life (HRQoL) and had less anxiety and depressive symptoms [11–13]. In most studies, the association of smoking with the mental health component of the HRQoL was stronger compared to that with the physical health component [13, 14]. Therefore, we hypothesized that depressive symptoms could be a mediator of the association between smoking and poorer HRQoL.

Over the last decades, factors associated with HRQoL among University students have been extensively studied [15, 16]. Furthermore, HRQoL is regarded as an important self-reported outcome measure and has been used in clinical studies focusing on subjective aspects of one’s health and functional status or well-being [17]. Even though the prevalence of chronic diseases among university students is low, and this population is considered overall healthy, the sensitivity of HRQoL measures allow for early detection of differences in health status between various students’ subgroups [15, 18].

The association of smoking with HRQoL among university students in low- and middle- income countries was rarely studied. The Republic of Serbia is classified as an upper middle-income country. Nevertheless, it is still in the process of political and economic transition [19]. In addition, Serbia is located in the Eastern Europe, a region with an overall high prevalence of tobacco smoking of > 25% [1]. In Serbia, about one third of students smoke cigarettes and this prevalence have been stable over time [20, 21].

There is scarcity of studies investigating a potential mediating effect of depression in the association between smoking and students’ HRQoL. The aim of this study was to assess the association between smoking and HRQoL among students in two different universities and the potential mediating effect of depression.

## Materials and methods

### Setting

This research included data from two cross-sectional studies, conducted at two Universities in the Republic of Serbia.

The University of Belgrade has around 90,000 students and consists of 31 schools. The University of Pristina temporarily seated in Kosovska Mitrovica (northern part) is a higher education institution of principal importance for the Serb population after the armed conflict at the turn of the 21^st^ century in Kosovo. The University of Pristina temporarily seated in Kosovska Mitrovica (Kosovska Mitrovica University) has approximately 8,000 students and consists of 10 schools. The Kosovska Mitrovica University is located in the northern Kosovo province, characterized by unstable political situation, in which neither the laws of the Republic of Serbia nor the laws of Kosovo are fully implemented. Accordingly, the law on smoking is not in effect, and smoking and second-hand smoking is common in public places. Additionally, evidence suggests that people who live in regions affected by the armed conflict smoke more compared to those who live in non-conflict areas [22]. Therefore, a discrepancy in the prevalence of smoking between Belgrade University and Kosovska Mitrovica University could be expected.

### Participants

A total of 1,624 students from the University of Belgrade and 514 students from the University of Kosovska Mitrovica who came to the Student Public Health Center for regular health check-ups were enrolled. Data were collected from April to June 2009 at the University of Belgrade [15, 23, 24] and from April to June in 2015 at the Kosovska Mitrovica University [25]. In all Universities in Serbia, regular annual health checks are mandatory for students in the first and third study year. Data from the Kosovska Mitrovica University were collected in a similar manner and during a similar period to mirror conditions and data collection from the University of Belgrade. Because of the difference in size of the total university student populations, with University of Belgrade having much larger population than Kosovska Mitrovica University, and similar length of data collection, the proportion of the study sample relative to the total student population was higher in the Kosovska Mitrovica University compared to University of Belgrade. The students were informed about the study purpose and received detailed explanations and definitions of certain terms. At any moment, at least one researcher was present at the area where students filled in the questionnaires. Participants in Belgrade provided signed informed consent for participation, while participants in the Kosovska Mitrovica provided verbal consent. Approval was obtained from the Ethics Committee of the School of Medicine University of Belgrade (approval no. 440/IV-1, issued on April 24, 2009) and the Ethics Committee of the School of Medicine Kosovska Mitrovica University (approval no. 01–503, issued on April 2, 2015). The response rate was 97.3% for Belgrade University and 98.6% for Kosovska Mitrovica University.

### Instruments

All study participants completed the socio-demographic questionnaire examining demographic information (age, gender, parental education, social status, place of birth and place of residence during study, type of school, presence of chronic diseases), habits and lifestyle (smoking, alcohol use, physical activity, eating habits). Smoking was defined as smoking at least one cigarette per day or smoking at least 100 cigarettes in a lifetime [26].

The HRQoL was assessed using a Short Form (SF) - 36 questionnaire (Administered in Serbian) [27]. The SF-36 questionnaire has previously been approved for use in Serbian language and showed good internal consistency [28]. The SF-36 consists of 36 questions that evaluate eight dimensions of health: physical functioning (PF), role functioning physical (RP), bodily pain (BP), general health (GH), vitality (VT), social functioning (SF), role functioning emotional (RE), and mental health (MH). In each domain, higher scores (range 0–100) reflect better self-perceived health per unit. Physical Composite Score (PCS) represents the mean value of scores of the first four domains, and the Mental Composite Score (MCS) represents the mean value of scores of the last four domains [27]. Finally, the Total score is calculated as mean of the Physical Composite Score (PCS) and the Mental Composite Score (MCS).

Beck Depression Inventory (BDI) was administered to evaluate the level of students’ depressive symptoms [29]. The BDI consists of 21 items, each scored on a scale of 0–3. The total BDI score ranges from 0 to 63 and represents the sum of grades for each item per unit. Cut-off ranges are as follows: 0–13 = no or minimal depression; 14–19 = mild depression; 20–28 = moderate depression; 29–63 = severe depression. The Serbian version of BDI, approved for use in Serbian language by the publisher (Pearson, San Antonio), demonstrated excellent internal consistency and test-retest reliability [30, 31].

### Data analysis

Data were analyzed using the SPSS for Windows, version 24. A probability level (p) of ≤0.05 was considered as statistically significant.

Measures of central tendency (mean and median) and variability (standard deviation and range) as well as relative numbers were used to describe the variables. Differences in the investigated parameters were assessed using: Chi-square test, t-test and ANOVA test with Tukey’s *post hoc* test.

To examine associations of smoking with HRQoL, we performed *hierarchical linear regression* analysis. Variables observed to be associated with HRQoL in the univariate linear regression analyses were included in the *multiple hierarchical linear regression* models. Prior to running regression models, multicollinearity was examined within the overall regression model using variance inflation factor of more than 2.0 as a cut-off for omission of collinear variables. Primary outcomes were PCS or MCS, and all the analyses were repeated for both outcomes. The independent variables were separated into five blocks (models) by covariates included.

Smoking was entered in the first block i.e. “Basic model”, allowing for unadjusted estimate. Next, in the “Socio-demographic model”, we added socio-demographic variables: age, gender, living arrangements and type of school. The third, “Behavior model”, included students’ habits: having breakfast, alcohol use and physical activity. In the fourth “Chronic disease model”, we added presence of chronic diseases and in the final “Depression model” BDI score was included. Because we were not able to include categorical independent variables directly in the hierarchical regression model, these were coded as dummy variables. This means that one category was defined as the reference, while all other categories within that variable were coded as separate binary variables. The effect estimates were presented as beta coefficients, with corresponding 95% confidence intervals.

To assess the associations of the independent variables with HRQoL score in more detail, we performed the quantile regression (QR) using Stata 14. We divided the entire range of SF-36 scores in five quantiles according to five cut-off points: 10^th^ (Q_10_), 25^th^ (Q_25_), 50^th^ (Q_50_), 75^th^ (Q_75_) and 90^th^ (Q_90_) percentile. The values of SF-36 scores per assigned quantile were examined as dependent variables. The QR provides an insight in the relationship between the independent variables at different points of conditional distribution and the outcome variable.

Preacher and Hayes’s bootstrapping method was used in the assessment of direct and indirect effect of smoking (dichotomous variable) on HRQoL (continuous variable) mediated by level of depression symptoms as measured by the BDI (continuous variable). The test was resampled 5,000 times. To correct for bias and skewness of bootstrap estimates, we used bias-corrected and accelerated bootstrap (BCa) interval. Bias-correction component accounts for the proportion of bootstrap estimates that are lower than the observed statistic. Acceleration component corresponds to skewness of the bootstrap distribution. Direct and indirect (i.e. pathway through a mediator variable) effect of smoking on HRQoL was considered statistically significant if the confidence interval did not include zero value. The effects have been adjusted for previously described variables in the hierarchical regression model.

The mediation model analysis was conducted in three steps. First, we confirmed that the independent variable (smoking) was associated with the dependent variable (HRQoL). Second, we confirmed that the independent variable (smoking) was associated with the variable that was considered a mediator (BDI). Third, we confirmed that the mediator (BDI) was associated with the dependent variable (HRQoL). Upon confirming that the mediator (BDI) was associated with the dependent variable (HRQoL), there is an assumption that the observed association between the independent (smoking) and the dependent variable (HRQoL) is greatly reduced or non-significant. If the association between the independent variable (smoking) and the potential mediator (BDI) was not observed, then the condition for mediation is not met [32].

## Results

The study included 1,624 (964 first year, 660 third year) students from the Belgrade University and 514 (264 first year, 250 third year) students from the Kosovska Mitrovica University. In both populations, females were more prevalent, 63.0% and 53.7%, respectively. Average age of students in both populations was 20.8 years (Table 1).

**Table 1:** Characteristics of the study populations.

Variables	Location of the University		p
	Belgrade (N = 1,624)	Kosovska Mitrovica (N = 514)	
Gender			**0.001**
*Male*	601 (37.0)	238 (46.3)	
*Female*	1023 (63.0)	276 (53.7)	
Age	20.83 (1.84)	20.75 (1.64)	0.323
Years of study			**0.001**
*First year*	964 (59.4)	264 (51.4)	
*Third year*	660 (40.6)	250 (48.6)	
Type of school			**0.001**
*Medical sciences*	195 (12.0)	238 (46.3)	
*Social sciences and humanities*	970 (59.7)	143 (27.8)	
*Natural sciences and mathematics*	81 (5.0)	14 (2.7)	
*Technology and engineering sciences*	378 (23.3)	119 (23.2)	
Living arrangements			**0.001**
*Home (with parents)*	758 (46.7)	140 (27.2)	
*Students’ dormitory*	180 (11.1)	189 (36.8)	
*Alone (in rented apartment)*	568 (35.0)	170 (33.1)	
*Other (with any family*, *hotel)*	115 (7.1)	15 (2.9)	
*Missing value*	3 (0.1)	/	
Having breakfast			0.651
*Yes*	931 (57.3)	289 (56.2)	
*No*	689 (42.4)	224 (43.6)	
*Missing value*	4 (0.3)	1 (0.2)	
Physical activity			0.372
*Yes*	1095 (67.4)	366 (71.2)	
*No*	486 (29.9)	147 (28.6)	
*Missing value*	43 (2.7)	1 (0.2)	
Chronic disease			0.308
*Yes*	201 (12.4)	55 (10.7)	
*No*	1423 (87.6)	459 (89.3)	
BDI score	6.70 (6.61)	6.40 (6.11)	**0.001**
Depression status			**0.001**
*No depression*	1407 (86.6)	453 (88.1)	
*Mild depression*	134 (8.3)	16 (3.1)	
*Moderate to severe depression*	83 (5.1)	17 (3.3)	
*Missing value*	/	28 (5.5)	
Smoking			0.253
*Ever*	428 (26.4)	149 (29.0)	
*Never*	1192 (73.4)	365 (71.0)	
*Missing value*	4 (0.2)	/	
Alcohol use			**0.001**
*Ever*	1314 (80.9)	351 (68.3)	
*Never*	279 (17.2)	163 (31.7)	
*Missing value*	31 (1.9)	/	
Smoking and Alcohol			**0.001**
*No smoking no alcohol use*	252 (15.5)	138 (26.8)	
*Only smoking*	26 (1.6)	25 (4.9)	
*Only alcohol use*	917 (56.5)	227 (44.2)	
*Both smoking and alcohol use*	394 (24.3)	124 (24.1)	
*Missing value*	35 (2.1)	/	
Smoking and Gender			**0.001**
*Male non-smokers*	455 (28.0)	162 (31.5)	
*Male smokers*	143 (8.8)	76 (14.8)	
*Female non-smokers*	737 (45.4)	203 (39.5)	
*Female smokers*	285 (17.5)	73 (14.2)	
*Missing value*	4 (0.3)	/	

Data are presented as number (%) or mean ± SD (Standard deviation). Bold values denote statistical significance (p<0.05). Missing value for age in Kosovska Mitrovica sample 2 (0.4%)

Differences in MCS (Belgrade 71.0 ± 18.5; Kosovska Mitrovica 75.6 ± 17.3, p = 0.001) and in PCS (Belgrade 80.0 ± 13.4; Kosovska Mitrovica 81.5 ± 13.6, p = 0.023) of HRQoL were observed between the two study population.

Students who ever smoked had lower MCS compared to students who had never smoked in their lifetime in both student samples (Belgrade 67.3 ± 19.9 vs. 72.3 ± 17.8, p = 0.001; Kosovska Mitrovica 70.5 ± 19.7 vs. 77.6 ± 15.8, p = 0.001) and the some results was obtained for PCS (Belgrade 78.3 ± 14.7 vs. 80.6 ± 12.9, p = 0.004; Kosovska Mitrovica 77.6 ± 15.9 vs. 83.2 ± 12.2, p = 0.001). Generally, scores of HRQoL related to mental health were lower compared with scores of domains related to physical health (Table 2).

**Table 2:** Differences in Mental Composite Score (MCS) and Physical Composite Score (PCS) of Health-related quality of life, Beck Depression Inventory scores and depression status between ever smokers and never smokers according to location of the University.

	Location of the University					
	Belgrade			Kosovska Mitrovica		
	Smoking Ever	Smoking Never	P	Smoking Ever	Smoking Never	p
Physical composite score	78.3 ± 14.7	80.6 ± 12.9	**0.004**	77.6 ± 15.9	83.2 ± 12.2	**0.001**
Mental composite score	67.3 ± 19.9	72.3 ± 17.8	**0.001**	70.5 ± 19.7	77.6 ± 15.8	**0.001**
BDI score	8.17 (7.63)	6.19 (6.14)	**0.001**	6.16 (8.45)	2.67 (4.57)	**0.001**
Depression status						
*No depression*	345 (80.6)	1058 (88.8)	**0.001**	117 (86.0)	336 (96.0)	**0.001**
*Mild depression*	47 (11.0)	87 (7.3)		8 (5.9)	8 (2.3)	
*Moderate to severe depression*	36 (8.4)	47 (3.9)		11 (8.1)	6 (1.7)	
				

Data are presented as number (%) or mean ± SD (Standard deviation). Bold values denote statistical significance (p<0.05)

In both student samples, students who ever smoked had higher BDI score compared to students who had never smoked (Belgrade 8.17 vs. 6.19, p = 0.001; Kosovska Mitrovica 6.16 vs. 2.67, p = 0.001). In both student populations, proportion of depression among students who ever smoked was higher compared to students who never smoked (Belgrade 19.4% vs. 11.2%, p = 0.001; Kosovska Mitrovica 14.0% vs. 4.0%, p = 0.001) (Table 2).

In the first four models of the hierarchical regression analysis, after adjustment for socio-demographic, behavioral and health factors, smoking remained associated with poorer PCS in students from the Kosovska Mitrovica and poorer MCS in both populations of students (Tables 3, 4 and 5). An exception from this pattern was observed for “Behavior” and “Chronic diseases model” in students from the Belgrade when PCS was examined (Table 6). The first four models explained 10.5–14.7% of variance (p = 0.001) for PCS and MCS score in both populations. In both samples, we observed that the magnitude of the association between smoking and HRQoL becomes weaker after adjustment for additional covariates.

**Table 3:** Hierarchical regression analysis of the association between students’ characteristics and Mental Composite Score among students from the Belgrade University.

Variable	Basic model			Socio-demographic model			Behavior model			Chronic diseases model			Depression model		
	B	95% CI	P	B	95% CI	p	B	95% CI	p	B	95% CI	p	B	95% CI	p
**Smoking** never vs. ever	5.21	3.12, 7.30	**0.001**	5.36	3.30, 7.42	**0.001**	3.65	1.57, 5.73	**0.001**	3.38	1.31, 5.44	**0.001**	1.12	-0.57, 2.80	0.193
**Age**				0.62	0.12, 1.13	**0.016**	0.49	0.01, 0.98	0.054	0.44	0.05, 0.92	0.081	0.11	-0.29, 0.51	0.598
**Gender** female vs. male				-5.98	-7.89, -4.08	**0.001**	-5.26	-7.15, -3.37	**0.001**	-5.15	-7.02, -3.28	**0.001**	-2.87	-4.39, -1.34	**0.001**
**Living arrangements**															
Home (with parents)				Reference category			Reference category			Reference category			Reference category		
Students’ dormitory				-3.43	-6.44, -0.42	**0.026**	-1.58	-4.56, 1.40	0.297	-1.80	-4.74, 1.15	0.232	-1.26	-3.56, 1.14	0.303
Alone (in rented apartment)				-3.01	-5.04, -0.98	**0.004**	-1.74	-3.75, 0.2	0.090	-1.68	-3.67, 0.31	0.097	-0.04	-1.66, 1.58	0.963
Other (with any family, hotel)				1.44	-2.25, 5.12	0.445	2.49	-1.12, 6.11	0.176	2.52	-1.06, 6.10	0.167	0.80	-2.11, 3.71	0.589
**Type of school**															
Medical sciences				Reference category			Reference category			Reference category			Reference category		
Social sciences and humanities				4.75	1.87, 7.62	**0.001**	4.71	1.89, 7.53	**0.001**	4.98	2.19, 7.77	**0.001**	4.30	2.03, 6.57	**0.001**
Natural sciences and mathematics				4.65	-0.11, 9.43	0.056	4.83	0.16, 9.49	**0.043**	5.62	1.00, 10.24	**0.017**	4.89	1.13, 8.64	**0.011**
Technology and engineering sciences				6.81	3.57, 10.07	**0.001**	7.08	3.90, 10.25	**0.001**	7.27	4.12, 10.41	**0.001**	5.54	2.98, 8.10	**0.001**
**Having breakfast** no vs. yes							-5.78	-7.61, -3.94	**0.001**	-5.85	-7.66, -4.04	**0.001**	-2.42	-3.91, -0.93	**0.002**
**Alcohol use** never vs. ever							1.01	-1.37, 3.38	0.405	0.99	-1.36, 3.34	0.407	0.26	-1.64, 2.17	0.787
**Physical activity** no vs. yes							-4.95	-6.93, -2.96	**0.001**	-5.04	-7.00, -3.07	**0.001**	-2.79	-4.39, -1.18	**0.001**
**Having chronic diseases** no vs. yes										8.00	5.34, 10.66	**0.001**	4.26	2.09, 6.44	**0.001**
**BDI score**													-1.61	-1.73, -1.50	**0.001**
**R**^**2**^	0.015			0.061			0.102			0.121			0.420		
**Sig. change in F**	**0.001**			**0.001**			**0.001**			**0.001**			**0.001**		

Bold values denote statistical significance (p<0.05)

**Table 4:** Hierarchical regression analysis of the association between students’ characteristics and Mental Composite Score among students from the Kosovska Mitrovica University.

Variable	Basic model			Socio-demographic model			Behavior model			Chronic diseases model			Depression model		
	B	95% CI	p	B	95% CI	p	B	95% CI	p	B	95% CI	p	B	95% CI	p
**Smoking** never vs. ever	6.81	3.50, 10.11	**0.001**	6.74	3.45, 10.04	**0.001**	5.88	2.55, 9.21	**0.001**	5.06	1.74, 8.37	**0.003**	0.77	-2.06, 3.60	0.593
**Age**				-0.67	-1.60, 0.25	0.154	-0.67	-1.58, 0.24	0.150	-0.50	-1.38, 0.43	0.298	-0.83	-1.59, -0.08	**0.031**
**Gender** female vs. male				-4.48	-7.50, -1.48	**0.004**	-3.32	-6.33, -0.32	**0.030**	-3.05	-6.06, -0.34	**0.048**	-2.16	-4.67, 0.36	0.093
**Living arrangements**															
Home (with parents)				Reference category			Reference category			Reference category			Reference category		
Students’ dormitory				0.02	-3.65, 3.70	**0.990**	-3.34	-2.44, 4.89	0.511	0.98	-2.65, 4.61	0.595	-0.85	-3.90, 2.19	0.581
Alone (in rented apartment)				-1.40	-5.53, 2.73	0.507	1.23	-3.89, 4.39	0.906	0.08	-4.02, 4.18	0.971	0.13	-3.30, 3.55	0.943
Other (with any family, hotel)				-11.47	-20.31, -2.62	0.011	0.25	-18.81, -1.37	0.023	-9.50	-18.14, -0.85	0.031	-4.57	-11.81, 2.67	0.216
**Type of school**															
Medical sciences				Reference category			Reference category			Reference category			Reference category		
Social sciences and humanities				1.88	-1.96, 5.72	0.337	1.93	-1.86, 5.71	0.317	1.40	-2.36, 5.16	0.465	0.89	-2.25, 4.03	0.576
Natural sciences and mathematics				6.54	-3.00, 16.07	0.179	8.71	-0.71, 18.14	0.070	8.21	-1.13, 17.55	0.085	6.30	-1.51, 14.10	0.113
Technology and engineering sciences				5.66	1.72, 9.59	**0.005**	6.41	2.51, 10.30	**0.001**	5.62	1.72, 9.51	**0.005**	3.15	-0.11, 6.42	0.058
**Having breakfast** no vs. yes							-5.90	-8.90, -2.90	**0.001**	-5.86	-8.83, -2.88	**0.001**	-2.35	-4.88, 0.18	0.068
**Alcohol use** never vs. ever							0.90	-2.28, 4.08	0.578	0.86	-2.29, 4.01	0.591	-0.00	-2.63, 2.63	0.998
**Physical activity** no vs. yes							-2.92	-6.19, -0.35	0.180	-2.92	-6.16, 0.32	0.077	-1.83	-4.54, 0.89	0.185
**Having chronic diseases** no vs. yes										7.92	2.98, 12.85	**0.002**	2.47	6.65, 0.19	0.248
**BDI score**													-1.56	-1.77, -1.35	**0.001**
**R**^**2**^	0.031			0.072			0.103			0.120			0.387		
**Sig. change in F**	**0.001**			**0.001**			**0.001**			**0.001**			**0.001**		

Bold values denote statistical significance (p<0.05)

**Table 5:** Hierarchical regression analysis of the association between students’ characteristics and Physical Composite Score among students from the Kosovska Mitrovica University.

Variable	Basic model			Socio-demographic model			Behavior model			Chronic diseases model			Depression model		
	B	95% CI	p	B	95% CI	P	B	95% CI	p	B	95% CI	p	B	95% CI	p
**Smoking** never vs. ever	5.23	2.66, 7.81	**0.001**	4.79	2.22, 7.35	**0.001**	3.95	1.36, 6.54	**0.003**	3.29	0.75, 5.83	**0.007**	0.56	-1.75, 2.87	0.635
**Age**				-0.57	-1.29, 0.15	0.120	-0.53	-1.23, 0.18	0.146	-0.40	-1.09, 0.30	0.181	-0.62	-1.24, -0.01	**0.049**
**Gender** female vs. male				-2.69	-5.04, -0.34	**0.025**	-1.65	-4.01, 0.72	0.172	-1.30	-3.61, 1.01	0.200	-0.73	-2.79, 1.33	0.485
**Living arrangements**															
Home (with parents)				Reference category			Reference category			Reference category			Reference category		
Students’ dormitory				-0.94	-3.81, 1.92	0.517	0.07	-2.78, 2.92	0.960	-0.22	-3.00, 2.57	0.879	-1.38	-3.87, 1.10	0.275
Alone (in rented apartment)				-3.52	-6.74, -0.30	**0.032**	-2.19	-5.41, 1.04	0.183	-2.39	-5.54, 0.76	0.136	-2.36	-5.16, 0.44	0.098
Other (with any family, hotel)				-12.03	-18.91, -5.14	**0.001**	-10.94	-17.72, -4.16	**0.002**	-10.24	-16.87, -3.61	**0.003**	-7.10	-13.03, -1.18	**0.019**
**Type of school**															
Medical sciences				Reference category			Reference category			Reference category			Reference category		
Social sciences and humanities				-0.11	-3.10, 2.88	0.942	-0.05	-2.99, 2.89	0.974	-0.67	-3.56, 2.21	0.646	-1.00	-3.57, 1.57	0.446
Natural sciences and mathematics				2.09	-5.33, 9.52	0.580	3.73	-3.61, 11.06	0.318	3.13	-4.03, 10.30	0.391	1.92	-4.47, 8.30	0.555
Technology and engineering sciences				3.21	014, 6.28	**0.040**	3.79	0.76, 6.83	**0.014**	2.86	-0.13, 5.84	0.061	1.29	-1.38, 3.96	0.344
**Having breakfast** no vs. yes							-4.00	-6.33, -1.66	**0.001**	-3.95	-6.23, -1.66	**0.001**	-1.71	-3.79, 0.35	0.104
**Alcohol use** never vs. ever							0.46	-2.01, 2.94	0.714	0.41	-2.00, 2.83	0.737	-0.14	-2.29, 2.02	0.301
**Physical activity** no vs. yes							-3.46	-6.01, -0.92	**0.008**	-3.46	-5.94, -0.97	**0.007**	-2.76	-4.98, -0.55	**0.015**
**Having chronic diseases** no vs. yes										9.39	5.60, -13.17	**0.001**	5.92	2.50, 9.35	**0.001**
**BDI score**													-0.99	-1.16, -0.82	**0.001**
**R**^**2**^	0.030			0.073			0.105			0.147			0.324		
**Sig. change in F**	**0.001**			**0.001**			**0.001**			**0.001**			**0.001**		

Bold values denote statistical significance (p<0.05)

**Table 6:** Hierarchical regression analysis of the association between students’ characteristics and Physical Composite Score among students from the Belgrade University.

Variable	Basic model			Socio-demographic model			Behavior model			Chronic diseases model			Depression model		
	B	95% CI	p	B	95% CI	p	B	95% CI	p	B	95% CI	p	B	95% CI	p
**Smoking** never vs. ever	2.52	1.00, 4.04	**0.001**	2.49	0.98, 4.00	**0.001**	1.24	-0.28, 2.78	0.110	1.01	-0.50, 2.52	0.189	-0.40	-1.71, 0.92	0.557
**Age**				0.41	0.05, 0.78	**0.029**	0.31	-0.05, 0.68	0.093	0.27	-0.09, 0.63	0.142	0.06	-0.25, 0.38	0.688
**Gender** female vs. male				-4.10	-5.50, -2.70	**0.001**	-3.55	-4.95, -2.16	**0.001**	-3.46	-4.82, -2.09	**0.001**	-2.0	-3.23, -0.84	**0.001**
**Living arrangements**															
Home (with parents)				Reference category			Reference category			Reference category			Reference category		
Students’ dormitory				-2.56	-4.78, -0.35	**0.023**	-1.25	-3.45, -2.16	0.262	-1.44	-3.60, 0.72	0.191	-1.10	-2.98, 0.78	0.250
Alone (in rented apartment)				-2.05	-3.54, -0.55	**0.007**	-1.15	-2.63, 0.32	0.126	-1.11	-2.56, 0.35	0.135	-0.09	-1.35, 1.18	0.896
Other (with any family, hotel)				0.50	-2.12, 3.20	0.720	1.23	-1.44, 3.89	0.367	1.24	-1.37, 3.87	0.350	0.19	-2.10, 2.47	0.874
**Type of school**															
Medical sciences				Reference category			Reference category			Reference category			Reference category		
Social sciences and humanities				2.15	0.03, 4.26	**0.046**	2.09	0.01, 4.16	**0.049**	2.32	0.28, 4.36	**0.026**	1.90	0.12, 3.68	**0.036**
Natural sciences and mathematics				3.89	0.39, 7.40	**0.029**	4.01	0.57, 7.44	**0.022**	4.70	1.31, 8.08	**0.007**	4.24	1.30, 7.19	**0.005**
Technology and engineering sciences				2.55	0.16, 4.94	**0.036**	2.72	0.38, 5.06	**0.023**	2.88	0.58, 5.19	**0.014**	1.82	-0.19, 3.82	**0.076**
**Having breakfast** no vs. yes							-3.81	-5.16, -2.47	**0.001**	-3.89	-5.21, -2.55	**0.001**	-1.75	-2.92, -0.58	**0.003**
**Alcohol use** never vs. ever							0.89	-0.86, 2.64	0.318	0.88	-0.84, 2.60	0.318	0.42	-1.07, 1.92	0.579
**Physical activity** no vs. yes							-3.82	-5.29, -2.36	**0.001**	-3.90	-5.34, -2.46	**0.001**	-2.51	-3.77, -1.25	**0.001**
**Having chronic diseases** no vs. yes										6.98	5.04, 8.93	**0.001**	4.67	2.95, 6.37	**0.001**
**BDI score**													-1.00	-1.09, -0.91	**0.001**
**R**^**2**^	0.006			0.039			0.077			0.105			0.323		
**Sig. change in F**	**0.001**			**0.001**			**0.001**			**0.001**			**0.001**		

Bold values denote statistical significance (p<0.05)

In the Belgrade sample, after addition of BDI in the full model, the association of smoking with HRQoL was lost. The “Depression model” explained 32.3% of variance of PCS and 42.0% of MCS (p = 0.001). This means that having more depressive symptoms was associated with poorer HRQoL and modified the association of smoking with HRQoL among students from Belgrade. Similarly, in Kosovska Mitrovica after inclusion of the BDI score in the final “Depression model”, the association between smoking and MCS and PCS was lost. The “Depression model” in the hierarchical regression analysis of the Kosovska Mitrovica sample explained 32.4% of variance for PCS and 38.7% for MCS (p = 0.001).

**Fig 1:**
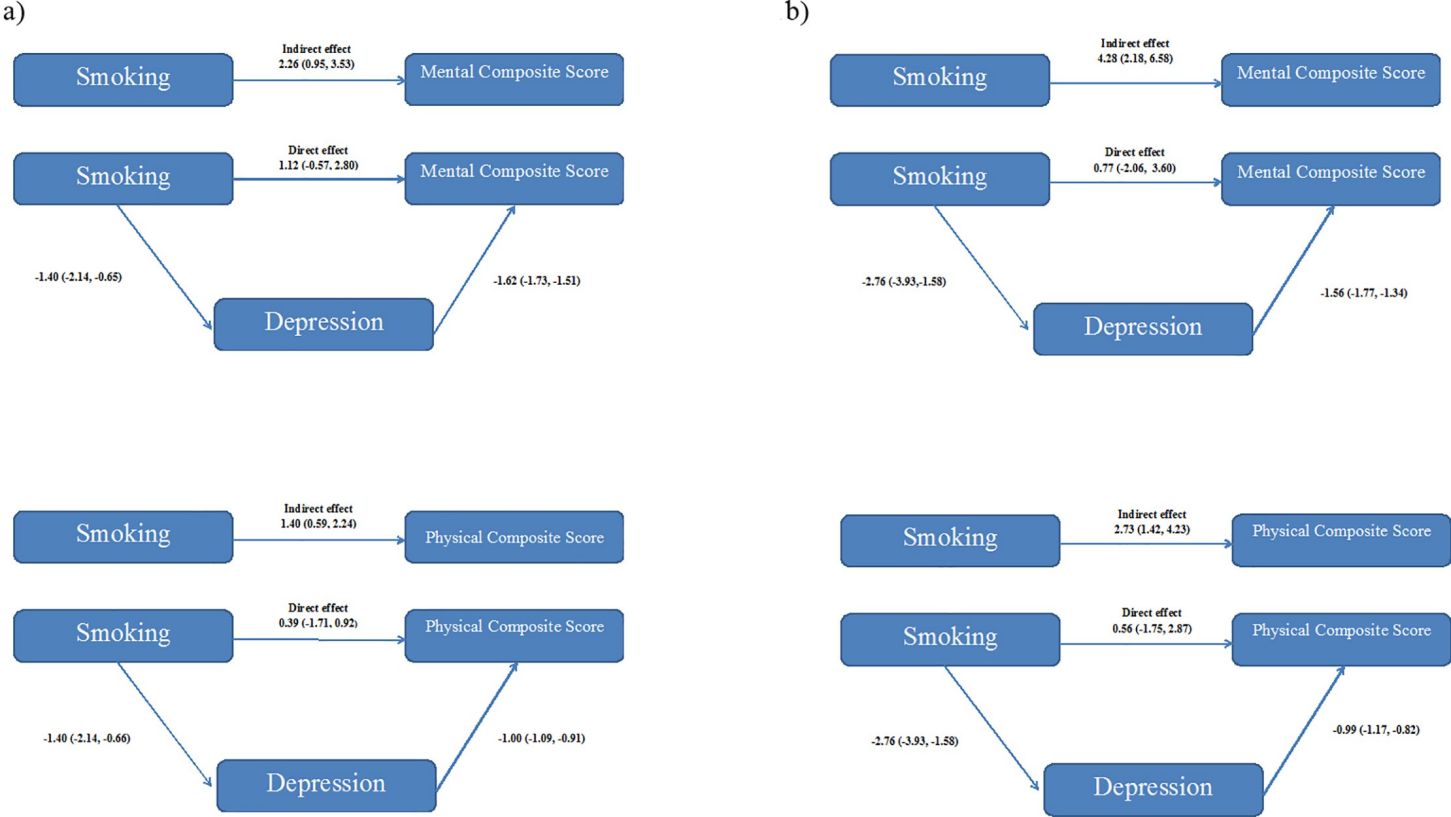
**Estimation of direct and indirect effects of smoking on Mental and Physical Composite Scores: 1a) students at the Belgrade University; 1b) students at the Kosovska Mitrovica University (**adjusted for age, gender, type of school, living arrangements, having breakfast, physical activity, alcohol use, having chronic diseases).

Preacher and Hayes’s method showed that a higher BDI was a mediator of the association between smoking and HRQoL. In both populations, smoking was associated with poorer MCS and PCS indirectly through higher level of depressive symptoms, while direct effect of smoking on HRQoL was not found (Belgrade: MCS direct effect 1.12, 95% CI -0.57, 2.80 and indirect effect 2.26, 95% CI 0.95, 3.53 and PCS direct effect 0.39, 95% CI -1.71, 0.92 and indirect effect 1.40, 95%CI 0.59, 2.24; Kosovska Mitrovica: MCS direct effect 0.77, 95% CI -2.06, 3.60 and indirect effect 4.28, 95%CI 2.18, 6.58 and PCS direct effect 0.56, 95% CI -1.75, 2.87 and indirect effect 2.73, 95%CI 1.42, 4.23) (Fig 1).

**Fig 2:**
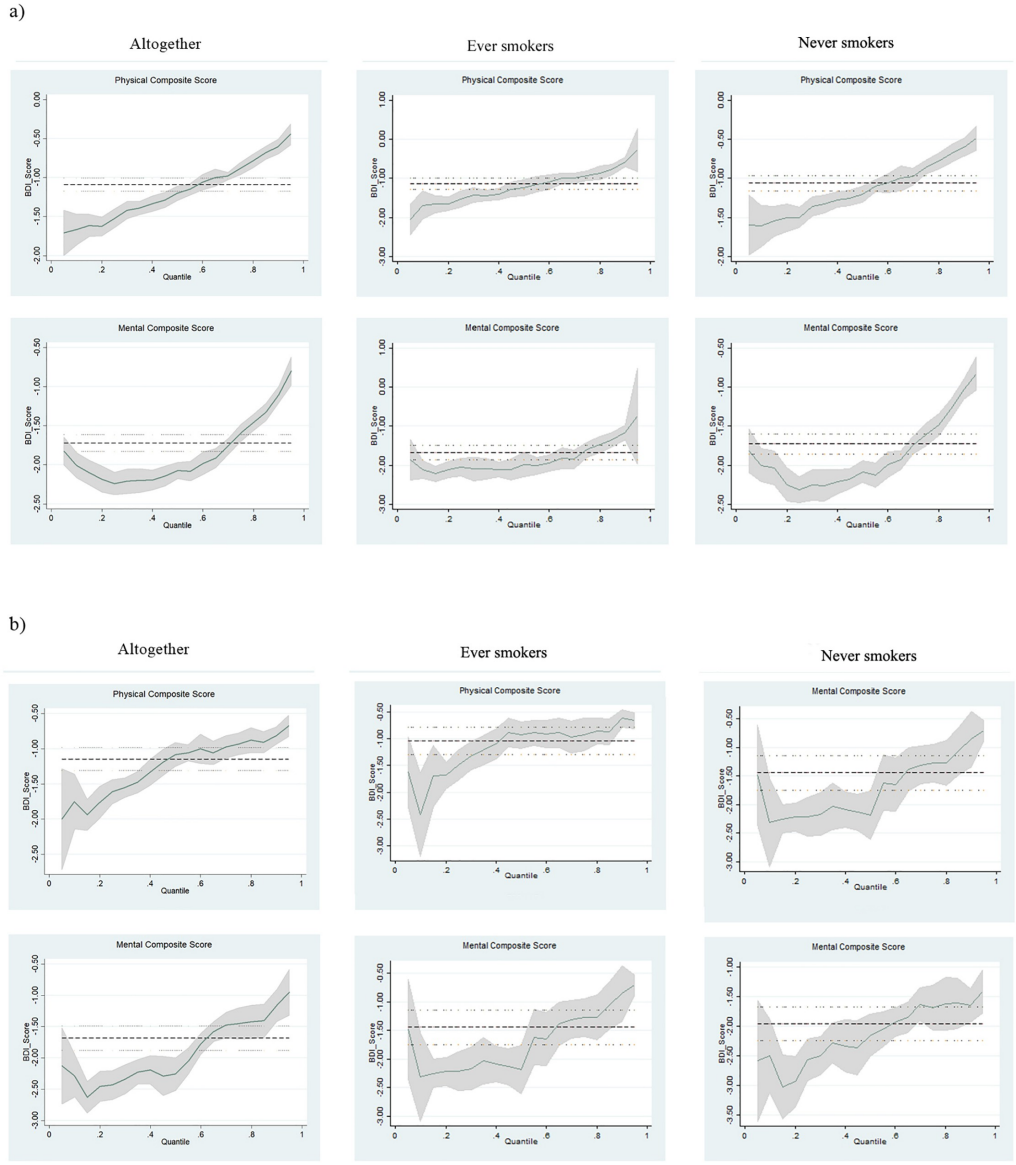
**Quantile regression analysis of the association between Beck Depression Inventory and Physical and Mental Composite Scores according to smoking status: 2a) students at the Belgrade University; 2b) students at the Kosovska Mitrovica University** (adjusted for age, gender, type of school, living arrangements, having breakfast, physical activity, alcohol use, having chronic diseases).

Additionally, quantile regression was performed to allow for a better insight in the association between study variables and conditional parts of the PCS and MCS (Tables 7 and 8). The BDI score was associated with poorer PCS and MCS across all quantiles in both populations. Magnitude of the association was particularly accentuated in lower quantiles (observed as lower half of tail of the outcome distribution in Fig 2)–Belgrade, β for: PCS -1.54 in Q_0.10_ vs. -0.58 in Q_0.90_; MCS -1.85 in Q_0.10_ vs. -1.07 in Q_0.90_ (p = 0.001); Kosovska Mitrovica, β for: PCS -1.86 in Q_0.10_ vs. -0.65 in Q_0.90_; MCS -2.34 in Q_0.10_ vs. -0.95 in Q_0.90_ (p = 0.001). The magnitude of the negative association between BDI score and lower quantiles of PCS and MCS was especially pronounced for never smokers and better MCS.

**Table 7:** Results of quantile regressions analysis of the associations between students’ characteristics and Mental Composite Score and Physical Composite Score of health-related quality of life among students from the Belgrade University.

	Q_0.10_		Q_0.25_		Q_0.50_		Q_0.75_		Q_0.90_	
	B	95% CI	B	95% CI	B	95% CI	B	95% CI	B	95% CI
**Mental Composite Score**										
Gender *female vs*. *male*	-3.67^**^	-6.46, -0.87	-3.46^**^	-5.94, -0.97	-2.11^*^	-3.97, -0.26	-2.51^**^	-4.28, -0.74	-2.04	-4.26, 0.17
Smoking ne*ver vs*. *ever*	-0.40	-3.48, 2.67	1.94	-0.79, 4.67	1.37	-0.67, 3.41	-0.10	-2.06, 1.84	0.34	-2.10, 2.77
Alcohol use ne*ver vs*. *ever*	0.48	-3.01, 3.96	0.31	-2.79, 3.40	0.97	-1.35, 3.28	0.46	-1.75, 2.67	-0.27	-3.04, 2.49
BDI score	-1.85^***^	-2.05, -1.64	-2.03^***^	-2.22, -1.85	-1.93^***^	-2.07, -1.79	-1.51^***^	-1.64, -1.38	-1.07^***^	-1.23, -0.90
**Physical Composite Score**										
Gender *female vs*. *male*	-3.48^*^	-6.56, -0.39	-2.29^*^	-4.32, -0.26	-1.69^**^	-2.96, -0.42	-124^**^	-2.21, -0.26	-0.87	-2.21, 0.46
Smoking ne*ver vs*. *ever*	-2.20	-5.60, 1.19	0.79	-1.45, 3.02	-0.26	-1.66, 1.14	-0.58	-1.65, 0.49	-0.38	-1.85, 1.07
Alcohol use ne*ver vs*. *ever*	-0.77	-4.62, 3.08	1.47	-1.07, 4.01	1.08	-0.51, 2.67	0.84	-0.38, 2.06	0.04	-1.62, 1.71
BDI score	-1.54^***^	-1.77, -1.31	-1.38^***^	-1.53, -1.23	-1.11	-1.20, -1.02	-0.82^***^	-0.89, -0.75	-0.58^***^	-0.68, -0.48

Bold values denote statistical significance (***p<0.001; **p<0.01; *p<0.05). Adjusted for age, type of school, living arrangements, having breakfast, physical activity, having chronic diseases

**Table 8:** Results of quantile regressions analysis of the associations between students’ characteristics and Mental Composite Score and Physical Composite Score of health-related quality of life among students from the Kosovska Mitrovica University.

	Q_0.10_		Q_0.25_		Q_0.50_		Q_0.75_		Q_0.90_	
	B	95% CI	B	95% CI	B	95% CI	B	95% CI	B	95% CI
**Mental Composite Score**										
Gender *female vs*. *male*	-0.29	-5.98, 5.39	-1.49	-5.33, 2.34	-1.44	-4.55, 1.66	-1.75	-4.35, 0.85	-2.55	-5.51, 0.41
Smoking ne*ver vs*. *ever*	-2.91	-9.29, 3.48	1.78	-2.52, 6.09	1.67	-1.82, 5.15	0.50	-2.43, 3.41	0.18	-3.15, 3.50
Alcohol use ne*ver vs*. *ever*	1.74	-4.20, 7.68	-1.48	-5.49, 2.52	1.62	-1.62, 4.86	1.09	-1.63, 3.81	2.13	-0.97, 5.22
BDI score	-2.34^***^	-2.82, -1.85	-2.06^***^	-2.38, -1.73	-1.84^***^	-2.11, -1.58	-1.36^***^	-1.58, -1.14	-0.95^***^	-1.20, -0.70
**Physical Composite Score**										
Gender *female vs*. *male*	2.36	-4.12, 8.83	-0.07	-3.16, 3.02	-1.60	-3.85, 0.66	-2.08	-4.51, 0.35	-1.87^***^	-3.06, -0.68
Smoking ne*ver vs*. *ever*	-0.56	-7.83, 6.70	0.97	-2.49, 4.44	-0.16	-2.69, 2.38	-0.08	-2.80, 2.65	0.35	-0.99, 1.68
Alcohol use ne*ver vs*. *ever*	-4.70	-11.46, 2.07	-0.63	-3.85, 2.60	1.12	-1.24, 3.48	1.58	-0.96, 4.12	1.88^***^	0.64, 3.12
BDI score	-1.86^***^	-2.41, -1.31	-1.51^***^	-1.77, -1.25	-0.96^***^	-1.15, -0.77	-0.83^***^	-1.03, -0.62	-0.65^***^	-0.76, -0.55

Bold values denote statistical significance (***p<0.001; **p<0.01; *p<0.05). Adjusted for age, type of school, living arrangements, having breakfast, physical activity, having chronic diseases

## Discussion

In this research we observed a strong association between smoking and poorer physical and mental health components of the health-related quality of life that persisted after adjusting for participants’ socio-demographic, behavioral and health factors. However, the associations with both PCS and MCS were explained by a mediation effect of the depressive symptoms. The mediating effect of depressive symptoms remained consistent across two student populations, six years apart, in two cities with socio-politically and economically diverse environment within the same country. Strong association between BDI score and PCS and MCS score was found across all five quantile scores.

In a sample from the Belgrade University, the PCS of students who never smoked was 2.3 points higher than PCS of students who ever smoked, and the MCS of students who never smoked was 5.0 points higher than MCS of students who ever smoked. In the sample from Kosovska Mitrovica, students who never smoked outscored ever smokers by 5.6 points in PCS and 7.1 points in MCS. The difference in HRQoL scores of 3 points or higher was shown previously to be clinically significant [33, 34]. Also, similar to previous studies [16, 35], we report a strong association of higher BDI score with lower PCS and MCS. Moreover, longitudinal BELLA study found presence of mental disorders as one of most important risk factors for lower HRQoL in children and adolescents [36].

In line with the previous studies, our results indicated that, in both populations, students who ever smoked had higher BDI score than students who never smoked in their lifetime [37, 38]. Also, in both populations, the association between smoking and HRQoL was lost after adjustment for the BDI score. Coste et al [39] reported that association of smoking with HRQoL was weaker but persisted when the BDI score was added in the regression model, which contrasts our findings. Depression has been reported as a stronger mediator between smoking and HRQoL among women while PCS was observed as an outcome [39]. Differences between our and study by Coste et al. could be possibly explained by differences in population characteristics such as older age of participants, classification of smokers as well additional potential modifiers and covariates accounted for in the current analysis [39].

We found that having more depressive symptoms mediated the association between smoking and poorer HRQoL. Our finding offers additional evidence to appreciate underlying mechanisms of the observed association between smoking and poorer HRQoL. Potential explanation may arise from the effects of nicotine on change in neurotransmitter activity that may lead to development of depression [40, 41]. It has been suggested that numerous chemicals in cigarette smoke interact with high-affinity nicotinic acetylcholine receptors and indirectly stimulate the release of dopamine, enabling, therefore, a sense of reward and positive reinforcement [41, 42]. This effect has also been observed to relieve feelings of depression and positively influence mood swings [41, 42]. Moreover, a study of Boden et al. found an unidirectional causal relationship between smoking and occurrence of depressive symptoms as well as that co-occurrence of smoking and depression cannot be explained by common genes and environment [6].

Impaired mental health is more prevalent in students compared to their peers from the general population and this is probably due to high academic demands [43–45]. The impairment of psychological well-being has been found to progress with the advancing years at school [46]. The study of Grøtan et al. reported a strong association of mental distress with poorer academic self-efficacy as well as with slower study progress [47]. The presence of depression may increase smoking rate in youth indirectly by increasing their vulnerability and acceptance of peer norms [5]. Smoking cessation may help to improve mental health and it is suggested that smoking cessation drugs may also have anti-depressive properties [9, 48]. Continuing stress during higher schooling has been suggested to contribute to the development of mental disorders and substance use disorders [49]. In students, behavioral response to stressors is ranked third, immediately after cognitive and emotional response [50].

In the quantile regression model, BDI score exhibited negative association with PCS and MCS across all quantiles in both student samples. This was especially pronounced among non-smokers who were more likely to have better mental health component of HRQoL, suggesting that non-smokers had better self-perceived mental health features. Thus, students who scored higher on the SF-36 scale tolerated depressive symptoms reasonably well, whereas students who reported poorer HRQoL have endured more depressive symptoms.

Our results provide a more thorough insight in the association between smoking and HRQoL and highlight a strong role of depressive symptoms in this association among university students. These findings suggest that combining the efforts of mental health service and substance abuse specialists could be relevant to enhance the promotion of healthy behavioral patterns and reduce the impact of depression on overall health [49]. Moreover, smoking might be regarded as a symptom of mental health disorders and a marker of increased risk for depression. Previous studies reported that people who have successfully quit smoking have better emotional and mental health stability and score better in the HRQoL measurements [41, 51]. On the other hand, anti-depressive drugs are used for smoking cessation [52]. Thus, our findings can be used to tailor intervention programs and strategies, particularly in populations with high prevalence of smoking and depression. A “Healthy setting” approach, such as Smoke Free Campus within the framework of Health Promoting Campus could combine programs and policies to improve mental health and prevent smoking [53]. As a result, incorporation of smoking cessation in depression treatment programs could yield better clinical outcomes [54]. Assessment of depressive symptoms using BDI could be a good tool to differentiate depression from other mental health conditions and help physicians make prompt and accurate assessment to foster safe and supportive academic environment.

In terms of study limitations, information bias should be considered, because data on smoking, alcohol use and other socio-demographic, behavioral and health factors were self-reported. Smoking status was dichotomized to smoking ever/smoking never and current smoking status, frequency and heaviness of smoking were not considered, therefore we cannot assess dose-response aspect of this association. In addition, cross-sectional study design did not allow for definitive conclusions on causality. Although we attempted to adjust for a number of potential confounders, there are possibly residual factors that were not included in the analysis as well as other unrecognized and unmeasured socio-demographic, behavioral and health factors that can modify effect estimate and introduce residual confounding. On the other hand, the findings were replicated in two populations which differ by time, place and characteristics. Because data from the University of Belgrade were collected in 2009, the study is limited by potential changes in tobacco use over the past decade [55]. Our study examined the association of cigarette smoking on HRQoL. In order to elaborate on this association, further research is warranted on other tobacco products, as well as quantity, frequency and duration of smoking or secondhand smoking.

In conclusion, the association of smoking with impairment of the HRQoL among students in two different settings was mediated by higher levels of depressive symptoms. Findings highlight the need for further research on the interaction between smoking, mental health and quality of life, with implications for prevention, diagnosis and treatment.
